# Biomonitoring of ochratoxin A, 2′R-ochratoxin A and citrinin in human blood serum from Switzerland

**DOI:** 10.1007/s12550-022-00456-0

**Published:** 2022-04-20

**Authors:** Alexandra Jaus, Peter Rhyn, Max Haldimann, Beat J. Brüschweiler, Céline Fragnière Rime, Judith Jenny-Burri, Otmar Zoller

**Affiliations:** Risk Assessment Division, Federal Food Safety and Veterinary Office (FSVO), Schwarzenburgstrasse 155, 3003 Bern, Switzerland

**Keywords:** Ochratoxin A, Citrinin, 2′R-Ochratoxin A, Ochratoxin alpha, Dihydrocitrinone, Human serum

## Abstract

Biomonitoring of mycotoxins and their metabolites in biological fluids is increasingly used to assess human exposure. In this study, biomarkers of ochratoxin A (OTA) and citrinin (CIT) exposure were determined in a large number of serum samples from healthy blood donors in Switzerland. In 2019, 700 samples from different regions were obtained. From 240 donors, a second sample (taken 2–9 months later) was available for analysis. Moreover, 355 blood donor samples from 2005 from all regions in Switzerland and 151 additional samples from the southern Swiss region of Ticino from 2005 could be analysed.

OTA, 2′R-ochratoxin A (2′R-OTA), ochratoxin alpha (OTα), CIT and dihydrocitrinone (DH-CIT) were analysed using validated targeted methods including precipitation and online SPE clean-up.

OTA and 2′R-OTA were frequently detected (OTA in 99%; 2′R-OTA in 51% of the tested samples). The mean concentration in all positive samples was 0.4 ng/mL for OTA and 0.2 ng/mL for 2′R-OTA. OTα was not detected in any sample above the limit of quantification (LOQ). In contrast to OTA, CIT and DH-CIT were only quantifiable in 2% and 0.1% of the samples, respectively. No significant trend was observed between the samples from 2005 and the more recent samples, but OTA concentrations were usually higher in serum samples from the southern Swiss region of Ticino and in males compared to females.

Our extensive data fit well within the framework of previously published values for the healthy adult European population.

**Supplementary information:**

The online version contains supplementary material available at 10.1007/s12550-022-00456-0.

## Introduction

Mycotoxins such as OTA and CIT are toxic secondary metabolites, which may be simultaneously produced by *Penicillium* and *Aspergillus* species. The occurrence of these two nephrotoxic mycotoxins in different commodities of the animal and human food chain raised concerns about the potential risks to human and animal health (Ali et al. 2015; EFSA 2012, 2020).

In Europe, cereals and cereal products are the main OTA sources (EC 2002; Sueck et al. 2019a). OTA has been classified in group 2B as a possible human carcinogen by IARC (1993). Recently, EFSA (2020) has published a new risk assessment on OTA in food. Here, margins of exposure (MOEs) were calculated for neoplastic and non-neoplastic effects from chronic dietary exposure to OTA, resulting in possible health concerns for certain consumer groups (e.g. infants).

In addition to OTA, the isomer 2′R-OTA is of relevance since it was found in all blood samples of coffee drinkers. The average concentration was half that of OTA (Cramer et al. 2015). 2′R-OTA is a diastereomer of OTA, which is not produced by fungi but is formed by isomerisation of OTA during thermal processing of contaminated food material such as coffee (Sueck et al. 2019a). In comparison with OTA, very little information on the toxicity of the degradation product 2′R-OTA is available (Sueck et al. 2019a).

For CIT, cereals were also described as the foods containing this mycotoxin in relevant levels (López Sánchez et al. 2017). However, CIT has also been reported in black olives, fermented meat products, cheese and apples (Silva et al. 2020). The European Food Safety Authority (EFSA) evaluated citrinin in 2012, but a full evaluation was not possible. Citrinin is nephrotoxic and a no-observed-adverse-effect level of 20 μg/kg body weight (bw) per day was identified from a 90-day study in rats. The derivation of a health-based guidance value was not considered appropriate, but a level of no concern for nephrotoxicity of 0.2 μg/kg bw per day was determined. Based on the available data, a concern for genotoxicity and carcinogenicity could not be excluded at the level of no concern for nephrotoxicity (EFSA 2012).

Risk assessment of mycotoxins usually uses data on food consumption and occurrence in the respective foods to estimate population exposure. However, this method cannot estimate individual intake (Gerding et al. 2014; Warensjö Lemming et al. 2020). Hence, biomarker-based methods are increasingly accepted as an efficient way to assess human exposure (Arce-López et al. 2020) as they allow to analyse physiological samples like urine or blood for each subject individually (Osteresch et al. 2017) and cover all dietary sources and exposure routes. Especially the suitability of OTA as biomarker has been studied in recent years: blood plasma or serum was recommended for the exposure evaluation due to the extensive binding to serum proteins and a long half-life of about 35 days in humans (Duarte et al. 2011; Fan et al. 2020; Märtlbauer et al. 2009; Studer-Rohr et al. 2000). Blood OTA levels are considered as suitable for estimating exposure of population groups (Commission for Human Biomonitoring of the German Environment Agency 2014); nevertheless, considerable inter- and intra-individual variations in the metabolism and excretion of OTA were observed (Studer-Rohr et al. 2000; Warensjö Lemming et al. 2020).

Consequently, according to Warensjö Lemming et al. (2020) and Duarte et al. (2011), individual daily dietary exposure assessment from blood or urine concentration remains difficult and understanding the human toxicokinetics and inter-individual differences needs more research.

Several sample preparation techniques and quantitation methods were described for OTA, CIT and their metabolites in various types of biomonitoring samples (Arce-López et al. 2020). Major challenges in these biomonitoring-based methods are usually low analyte concentrations and matrix interferences during analysis (Osteresch et al. 2017).

The primary aim of this work was to investigate serum levels in adults in Switzerland for the frequently detected mycotoxins OTA and 2′R-OTA as well as CIT, which has been detected in plasma samples in Germany (Blaszkewicz et al. 2013). The metabolites OTα and DH-CIT were included into the analysis. Furthermore, temporary trends as well as possible associations between serum levels and sex or regions were investigated. For this study, a validated targeted analytical method was developed using online SPE clean-up followed by HPLC-MS/MS detection. This approach combines suitability for large sample numbers with high sensitivity.

## Materials and methods

### Serum samples and ethical permits

An overview on the serum samples is given in Table 1. The ethical approval for the 2019 study was obtained from the Cantonal Ethic Committee Bern (No 2018-02,137). All participants providing blood samples left written informed consent prior to participation.

**Table 1:** Characteristics of the investigated human serum samples from Switzerland

Designation of sub-study	Sampling Date	Sample number	Origin
2019			
**2019 Phase I**	June–August 2019	n = 700	Blood donors from all regions (including 100 samples from Ticino)
**2019 Phase II**	October 2019–March 2020	n = 240	Participants of 2019 Phase I re-sampled after 2–9 months
2005			
**2005**	May–July 2005 or May–September 2006	n = 355	Balanced subsample of 1847 donors from 9 blood bank centres of all regions (Burri et al. 2008) with comparable age and sex structure as the whole collective (including 40 samples from Ticino)
**2005 TI + **		n = 151	All further samples from Ticino of the study by Burri et al. (2008)

The 2005 study was approved by the Cantonal Ethic Committees in the respective Swiss cantons. All samples were transported under dry ice and stored at -40 °C until further use.

### Chemicals and reagents

Acetonitrile was of LC-MS purity (Biosolve Chemie Brunschwig, Basel, Switzerland). Methanol UHPLC-MS was purchased from Scharlab (Sentmenat, Spain). Ultrapure laboratory water, further referred to as water, was obtained from a Milli-Q IQ7000 ultra-water purification system (Merck-Millipore, Zug, Switzerland). Formic acid for LC-MS, sodium chloride (for molecular biology, ≥ 99%), ortho-phosphoric acid 85% (suprapur^R^), ammonium acetate (for mass spectrometry), sodium acetate trihydrate (for analysis), L-ascorbic acid (puriss. p. a.), ethylenedinitrilotetraacetic acid (for analysis), acetic acid (puriss. p. a.) and newborn calf serum were from Merck Sigma-Aldrich (Buchs, Switzerland). OTA, CIT, OTα, ^13^C-labelled OTA (^13^C_20_-OTA) and ^13^C-labelled CIT (^13^C_13_-CIT) were purchased from Romer Labs (Butzbach, Germany). DH-CIT was obtained from AnalytiCon Discovery (Potsdam, Germany) and 2′R-OTA from Aokin (Berlin, Germany). All standards were stored at -20 °C. Stock solutions were prepared at concentrations of 1000 ng/mL in acetonitrile and stored at 5 °C in the dark until further use. Working solutions with 50 ng/mL of all analytes were diluted from these stock solutions and stored at 5 °C for 1 month at most. Polypropylene pipet tips for Gilson microman (Gerber Instruments, Effretikon, Switzerland) were used for all volumetric transfers.

β-Glucuronidase/arylsulfatase (β-Gluc/ArylS) from *Helix pomatia* (with specific activity 5.5 U/mL β-glucuronidase, 2.6 U/mL arylsulfatase at 38 °C) was purchased from Merck Sigma-Aldrich (Buchs, Switzerland) and used with hydrolysis buffer (6.8 g sodium acetate trihydrate, 500 mg ascorbic acid, 50 mg ethylenedinitrilotetraacetic acid in 50 mL water, adjusted to pH 5.0 with acetic acid) for the enzymatic treatment of a number of serum samples.

### Sample preparation

A 200 µL aliquot of serum was transferred into a 2-mL Eppendorf safe-lock tube (Huberlab, Aesch, Switzerland), and 600 µL of extraction solvent (ortho-phosphoric acid 85%/methanol/water/sodium chloride (10/63/27/1, w/w/w/w)) containing 0.5 ng/mL of internal standards was added. The tubes were vortex-mixed for 60 s and left for 10 min at room temperature. Thereafter, the tubes were centrifuged at 42,000 × g and 4 °C for 10 min (Eppendorf 5417 R, Vaudaux-Eppendorf, Schönenbuch, Switzerland). The supernatant was transferred into a polypropylene vial (Infochroma, Goldau, Switzerland) and used for analysis. For standards and quality control samples, spike solution was directly added to 200 µL of newborn calf blank serum with no detectable concentrations of the analytes.

### Enzymatic cleavage of conjugates

For some samples with elevated OTA, an enzymatic treatment similar to the procedure described by Muñoz et al. (2010) was performed to possibly increase the OTα concentration: 250 µL of serum was mixed with 25 µL of hydrolysis buffer solution and 5 µL of enzyme. The samples were kept at 37 °C overnight prior to sample preparation of a 200-µL aliquot.

### HPLC-MS/MS conditions

#### Online SPE-HPLC system and conditions

A Spark Symbiosis-System (Axel Semrau, Sprockhoevel, Germany) was used with Oasis HLB on-line SPE cartridges (10 × 2 mm, Waters, Eschborn, Germany). The cartridges were cleaned and conditioned with 1.0 mL acetonitrile containing 0.5% formic acid (at 1.0 mL/min) and 1.0 mL methanol (at 1.0 mL/min) followed by equilibration with 1.0 mL water (at 0.5 mL/min). Next, 60 µL of sample solution was loaded onto the cartridge using 0.9 mL water/extraction solvent (2/6, v/v, at 0.2 mL/min). After completion of SPE loading, the cartridge was switched into elution position and the analytes were transferred onto the analytical column with 0.35 mL acetonitrile containing 0.5% formic acid (at 0.1 mL/min).

During elution of the current cartridge, the next cartridge was placed in the conditioning position and underwent conditioning and loading.

Autosampler needle rinsing was performed as follows: 500 µL water/methanol/acetonitrile 8/1/1 (v/v/v) followed by 500 µL water/methanol 1/1 (v/v) with 0.1% phosphoric acid 85% and finally 500 µL of water.

For chromatographic separation, a Nucleodur C18 Gravity-SB column (3 μm, 2.0 × 50 mm, Macherey-Nagel, Oensingen, Switzerland) equipped with a guard column (1.8 µm, 2.0 × 4.0 mm) of the same column material was used. The column temperature was 40 °C and the flow rate 0.6 mL/min. Eluent A was water with 5 mM ammonium acetate and eluent B methanol/acetonitrile/water (50/45/5, v/v/v) with 5 mM ammonium acetate. The following gradient program applied: 0 min, 1% B; 3:30 min, 1% B; 3:36 min, 35% B; 4:30 min, 40% B; 5:00 min, 65% B; 7:00 min, 65% B; 7:06 min, 1% B; 7:40 min, 1% B. A diverter valve was installed between column and mass spectrometer and only from 4:30 to 6:30 min the HPLC eluted into the mass spectrometer.

#### MS/MS-system and conditions

An API 5000 (Sciex, Brugg, Switzerland) with electrospray ionisation (ESI) in negative mode was used and scheduled multiple reaction monitoring (sMRM) was applied (MRM detection window 40 s, target scan time 0.5 s). ESI-source parameters were optimised and set to a source temperature of 600 °C, ionisation voltage -3000 V, curtain gas 25 units, collision gas 8 units, GS1 (nebuliser gas) 50 units and GS2 (heater gas) 50 units.

Two MRM transitions with optimum signal-to-noise ratios were monitored per analyte; the one with the highest signal intensity was selected as quantifier. Supplementary Table S1 lists detailed HPLC-MS/MS parameters, and supplementary Figure S1 shows a typical HPLC-MS/MS chromatogram of a spiked serum recovery sample with the quantifier transition of each analyte. OTA and 2′R-OTA were baseline-separated, and the CIT peak showed only acceptable tailing. Chromatographic retention of the first eluting compound OTα was adjusted to > 5 min in order to discard remaining sample matrix and online SPE-reagents by a diverter valve.

#### Calibration

Calibration solutions were freshly prepared by dilution of the working solutions on every measurement day. Matrix-induced signal suppression and enhancement were observed by several authors during analysis of mycotoxins in serum (Osteresch et al. 2017; Silva et al. 2020). Therefore, matrix-matched calibration was applied: For every analyte, a 6-point calibration curve, corresponding to a range of 0.0 to 1.0 ng/mL, was generated by spiking calibration solution mix into blank calf serum followed by sample preparation. For 2′R-OTA, OTα and DH-CIT, no specific isotope-labelled internal standard was available; therefore, ^13^C_20_-OTA was used as internal standard for 2′R-OTA and OTα while ^13^C_13_-CIT was used for DH-CIT.

If a sample contained a concentration above the calibration range, it was diluted with blank calf serum prior to sample work-up or a further calibration point with 2 ng/mL was used.

#### Method validation

The established method was validated as described below by determining the linearity, limit of detection (LOD), LOQ, recovery and precision to ensure its sensitivity, accuracy and repeatability.

A series of 7 blank calf serum samples was spiked with 0.0 to 2.0 ng/mL of all analytes followed by standard sample preparation procedure as described above. Calibration curves (1/x weighted) were created for each analyte by plotting the area ratios versus the concentration ratios. The sensitivity was assessed in these samples by determining the LOQ (signal-to-noise ratio (S/N) of 10:1) and LOD (S/N of 3:1). The recovery, repeatability and inter-day precision tests were all performed in five independent replicates using blank calf serum samples spiked with low or high concentration levels (see supplementary Table S2) and matrix-matched calibration. Recovery was calculated by comparing the measured concentration using the matrix-matched calibration curves with the spiked concentration of each analyte. The relative standard deviations (RSDs) determined in these samples (n = 5) were used for evaluation of the intra-day precision, whereas the results of fortified recovery samples on 2 different days (n = 10) were used for inter-day precision. Moreover, the injection repeatability of the online SPE-HPLC-MS/MS part was validated by injecting the same sample solution 5 times. Detailed data of spiked concentrations, results and RSDs are shown in supplementary Table S2.

Stability testing of final sample solutions was done by storing the vials at 10 °C in the dark (autosampler conditions) and re-analysing them after 2, 4 and 6 h. Reproducibility was determined for OTA and CIT by analysing an internal human reference pool serum sample (naturally containing OTA and spiked with CIT) with every analytical series.

#### Quality control

A limited stability of the sample solutions of 4 h maximum was observed; therefore, samples had to be prepared in sub-sequences. To ensure correct preparation and analysis, a quality control sample (blank calf serum fortified with 0.5 ng/mL of all analytes) was worked up with every sub-sequence. To avoid any analytical bias, 2019 phase I and phase II samples from the same blood donor were always analysed as subsequent samples within the same analytical series. Moreover, as described above an internal reference sample was analysed for OTA and CIT every day of analysis.

#### Statistical analysis

Statistical analyses for OTA were performed using Systat software version 13 (SigmaPlot, Düsseldorf, Germany). For data analysis in terms of sampling time, sex and region, differences between different subgroups were compared with z-tests. Serum OTA concentrations below the LOQ were set to one-half of the LOQ. The test was considered as statistically significant when the p value was ≤ 0.05.

For specific comparison of OTA 2019 phase I and II results, a paired Wilcoxon signed-rank test was performed. Four samples with at least 1 value < LOQ were not considered here. The test was considered as statistically significant when the p value was ≤ 0.05. This nonparametric test was chosen because no normal distribution of the differences between phase I and II was observed.

## Results and discussion

A fast and robust multi-mycotoxin method for human serum with online SPE-HPLC-MS/MS analysis in negative electrospray ionisation mode and scheduled multiple reaction monitoring was developed and applied to more than 1400 human serum samples.

### Optimisation of sample work-up and HPLC-MS/MS conditions

Several authors reported interaction and even formation of stable complexes of human serum albumin and mycotoxins at pH close to 7 (Poor et al. 2015; Sueck et al. 2018). Therefore, on the basis of the acid extraction reagent used by Zimmerli and Dick (1995), a reagent composed of ortho-phosphoric acid, sodium chloride, methanol and water was applied. This reagent ensured the release of possibly adsorbed mycotoxins as well as protein denaturation in combination with slow precipitation of small particles, thus avoiding the enclosure of mycotoxins into the sediment of particles.

Optimisation of sample preparation and SPE-conditions was performed with offline cartridges before switching to online-SPE. It was observed that complete elution of all compounds from the cartridges could only be achieved with acetonitrile containing at least 0.5% formic acid. Starting with the LC-MS/MS method of Osteresch et al. (2017), an eluent without acetic acid but with ammonium acetate was chosen, and thus, sufficient sensitivity for all compounds with ESI in negative ionisation mode was observed. A MS-method without polarity switching was very favourable for developing a method with short run time allowing high throughput. Nevertheless, the baseline separation of OTA/2′R-OTA was essential for quantification due to the same fragmentation pattern of both isomers. The main challenge was to ensure only moderate tailing of the CIT peak while achieving baseline separation of OTA/2′R-OTA. Thanks to online SPE, it was possible to inject 60 µL of sample solution without requirement of any error-prone concentration steps. The use of a specific needle rinsing procedure was important, especially for CIT to minimise carryover and contamination. After analysing standards with high CIT concentration, a subsequent injection of methanol was performed to avoid any carryover.

### Validation

Validation was carried out by determining linearity, matrix effects, recovery, LOD and LOQ in blank calf serum matrix (supplementary Table S2). Average recovery rates of 70 to 120% and regression coefficients r^2^ of 0.9921 to 0.9964 by linear regression were achieved for all validation experiments. As expected, higher RSDs were generally observed for the compounds without “specific” internal standard. For the main analytes OTA, 2′R-OTA and CIT, the LOQ values obtained with this new method were comparable to or in the case of CIT (LOQ 0.05 ng/mL) even better than those previously published (Degen et al. 2018; Malir et al. 2019; Osteresch et al. 2017; Ouhibi et al. 2020). For OTA, the LOQ of 0.1 ng/mL obtained here was largely sufficient since in general values above this level were determined. However, for DH-CIT and OTα disturbing peaks and a comparatively high noise level were observed, which led to higher LOQs (0.25 ng/mL) than for the other reported compounds. The long-term reproducibility was determined with an in-house human reference pool serum sample analysed once in every analytical series, resulting in a long-term standard deviation of 16.2% for OTA (0.24 ng/mL, n = 35) and 10.0% for CIT (0.13 ng/mL, n = 35). Regarding inter-day repeatability, comparatively high standard deviations (approx. 15%) were observed at high recovery level for all compounds quantified with ^13^C_20_-OTA. Since the repeatability RSD of OTA was still in the range of the long-term reproducibility and both values were comparable with the OTA data published by Warensjö Lemming et al. (2020), this was accepted.

In summary, the developed method achieved reliable validation parameters for mycotoxin detection in human serum. It accomplished a stable and robust quantification performance by use of matrix-matched calibration in combination with commercially available isotopically labelled standards, and was considered fit for purpose.

### Biomonitoring of serum samples

Since recovery rates > 90% for OTA, CIT, DHCIT and OTα were reported by Osteresch et al. (2017) for dried serum spots after storage at -18 °C for 24 weeks, we assume sufficient analyte stability for our serum samples from 2019 stored in vials at -40 °C for a maximum of 16 months. This is supported by the results of the internal human reference pool serum sample where no decreasing analyte concentrations over time were observed.

There remains an uncertainty for the results of the samples from 2005, but since no obvious differences to the results from 2019 were observed, there is no indication for insufficient analyte stability in serum.

A general overview of the results for OTA, 2′R-OTA, CIT, DH-CIT and OTα in the 2019 and 2005 serum samples is given in Table 2 and Table 3. For comparison, the OTA results from Zimmerli and Dick (1995) are also listed in Table 2.

**Table 2:** Overview of OTA and 2′R-OTA serum levels of blood donors in Switzerland — results from 2019 (phase I n = 700, phase II n = 240), 2005 (n = 355) and 1995 (n = 366)

	% ≥ LOQ	Mean^a^ [ng/mL]			Median [ng/mL]			Maximum [ng/mL]
		**All**	Female	*Male*	**All**	Female	*Male*	
**OTA**								
2019 Phase I^b^	98.7	**0.42**	0.33	*0.50*	**0.27**	0.26	*0.30*	23.0
2019 Phase II^c^	99.2	**0.38**	0.36	*0.40*	**0.28**	0.27	*0.31*	1.73
2005	99.2	**0.40**	0.32	*0.47*	**0.28**	0.28	*0.28*	14.6
1995^d^	100	**0.39**	0.27	*0.49*	**n.d.**	0.24	*0.30*	6.02
**2′R-OTA**								
2019 Phase I	48.1	**0.23**	0.24	*0.22*	**n.d.**	n.d.	*n.d.*	1.31
2019 Phase II	52.1	**0.24**	0.24	*0.23*	**0.10**	0.11	*0.10*	1.15
2005	50.7	**0.20**	0.20	*0.20*	**0.10**	0.10	*0.10*	0.98

^a^Mean is calculated in positive samples, excluding values < LOD

^b^1 extreme value of approx. 700 ng/mL was excluded

^c^Phase II samples: 240 samples taken 2–9 months later from Phase I donors

^d^data from Zimmerli and Dick (1995)

n.d. not determined

**Table 3:** Overview of CIT, OTα and DH-CIT serum levels of blood donors in Switzerland — results from 2019 (phase I n = 700, phase II n = 240) and 2005 (n = 355)

	% ≥ LOQ	Mean^a^ [ng/mL]	Maximum [ng/mL]
**CIT**			
2019 Phase I	2.1	0.09	5.70
2019 Phase II^b^	1.3	0.06	0.85
2005	2.5	0.05	0.54
**OTα**			
2019 Phase I	0	n.d.	< LOQ
2019 Phase II	0	n.d.	< LOQ
2005	0	n.d.	< LOQ
**DH-CIT**			
2019 Phase I	0.1	n.d.	< LOQ
2019 Phase II	0	n.d.	< LOQ
2005	0.1	n.d.	0.32

^a^Mean is calculated in positive samples, excluding values < LOD, values between LOD and LOQ were replaced with ½ LOQ (CIT only)

^b^Phase II samples: 240 samples taken 2–9 months later from phase I donors

n.d. not determined

Due to low occurrence median not determinable for any analyte

If comparing our results with other studies, it is important to consider that Korn et al. (2011) determined a maximum difference of approximately 20% between OTA concentrations in serum and plasma, depending also on the anticoagulant used for plasma stabilisation. The concentration ratio of OTA in serum to whole blood was determined as approximately 2:1 (Korn et al. 2011; Zimmerli and Dick 1995). For 2′R-OTA, CIT, DH-CIT and OTα, to the best of our knowledge, no such ratios were determined but values similar as for OTA were assumed, since they were in good agreement with the volume ratios (serum to plasma 1:1 and serum to whole blood 1:2).

### OTA-Results

#### Results and trends in Switzerland over the last three decades

The 700 samples from the 2019 phase I sub-study (Table 1) provided valuable insight into the current human OTA serum levels in Switzerland.

**Fig. 1:**
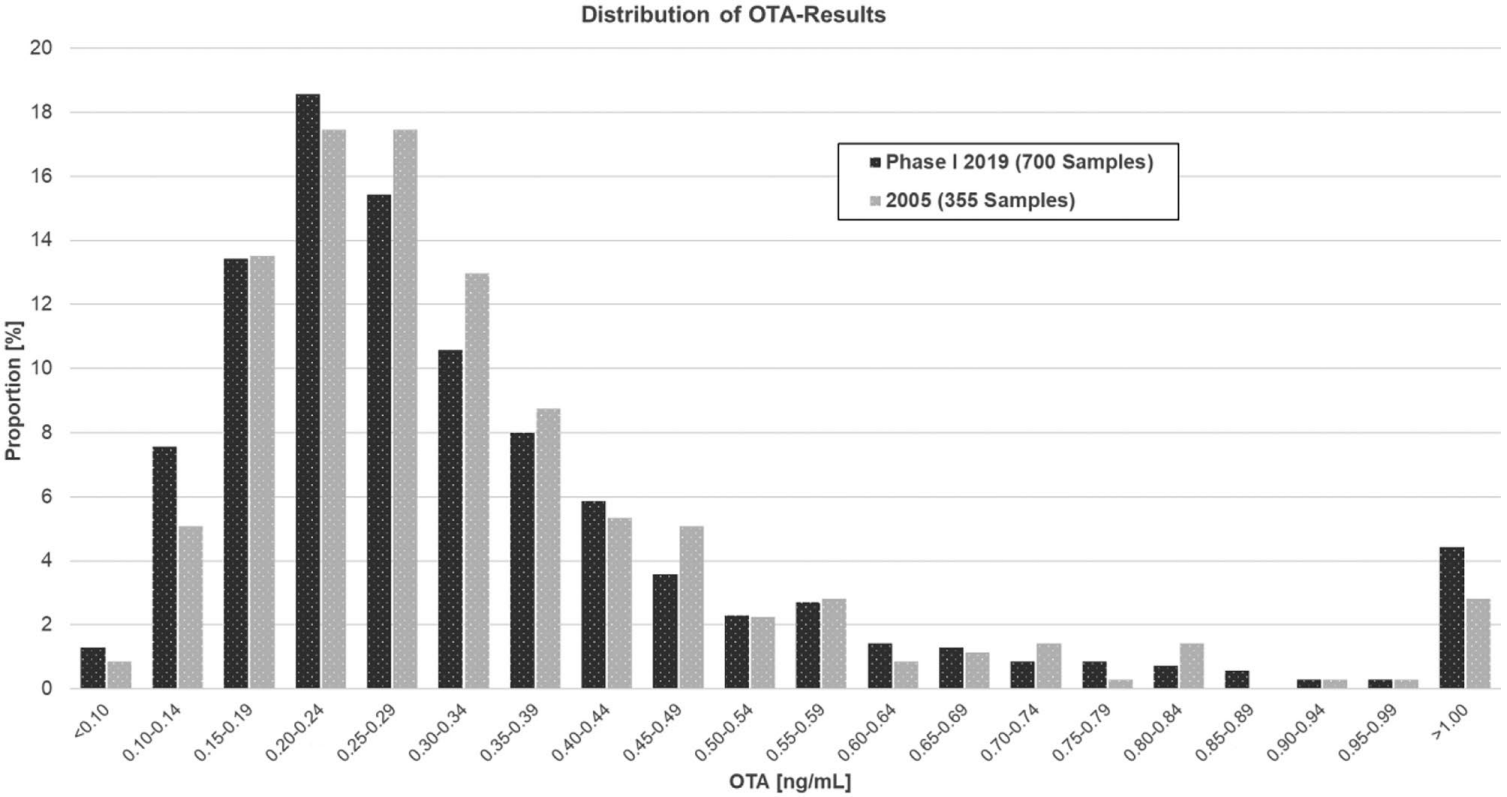
Distribution of OTA serum levels of blood donors in Switzerland — results from 2019 and 2005

As illustrated in Fig. 1, the OTA results showed a right-skewed distribution. Not normally distributed values have already been described by several authors (Ali et al. 2014; Fan et al. 2020; Medina et al. 2010; Zimmerli and Dick 1995). It was not possible to fit a parametric distribution to the data set neither for the OTA nor for the log OTA results, which is in agreement with the observation by Zimmerli and Dick (1995). Although the data were not normally distributed, mean values were calculated for the purpose of comparison within our study and with other publications.

Very similar detection rates, means and medians were observed for the samples from 2019 phase I, 2005 and 1995 (all results in Table 2) in spite of different sample preparation (online SPE instead of liquid-liquid extraction and immunoaffinity column clean-up 1995) and analytical technique (HPLC-MS/MS instead of HPLC-FLD 1995).

Furthermore, a very similar right-skewed distribution of the results can be observed at both sampling points 2019 and 2005 (Fig. 1). Multiple z-tests using Bonferroni correction of the 2019 phase I and 2005 results revealed no significant differences between sampling time points (p = 0.99).

#### Determinants on serum levels

Zimmerli and Dick (1995) reported elevated serum OTA concentrations for people, especially males living in the southern Swiss region of Ticino.

Therefore, particular attention was paid to analyse a sufficiently large sample number of this region: For 2019, 100 phase I samples were available (Table 1). For 2005, the Ticino was originally represented with 40 out of the total 355 samples. Since this sample number may be too low for sufficient statistical evidence, all further 151 available samples from Ticino 2005 (Burri et al. 2008) were analysed (2005 TI + samples in Table 1) in order to obtain more stable estimates and a better comparison with the other regions.

Detailed OTA results for all samples from Ticino are given in Table 4. It can be observed that both mean and median were higher than the average values in Switzerland at all measurement times. A z-test of the 2019 phase I and 2005 results including the 2005 TI + samples revealed that significant higher levels were observed in Ticino compared to other regions in Switzerland (p < 0.05).

**Table 4:** Overview of OTA and 2′R-OTA serum levels of blood donors in Ticino — results from 2019 (Ticino samples from phase I n = 100, 42 female, 58 male donors), 2005 (all samples from Ticino available: n = 40 + 151, 55 female, 136 male donors) and 1995 (n = 116, 44 female, 72 male donors)

	% ≥ LOQ	Mean^a^ [ng/mL]			Median [ng/mL]			Maximum [ng/mL]
		**All**	Female	*Male*	**All**	Female	*Male*	
**OTA**								
2019 Phase I^b^	100	**0.86**	0.40	*1.19*	**0.35**	0.31	*0.39*	23.0
2005	99.5	**0.55**	0.40	*0.61*	**0.40**	0.33	*0.43*	7.29
1995^c^	100	**0.65**	0.30	*0.87*	**n.d.**	0.29	*0.42*	6.02
**2′R-OTA**								
2019 Phase I	54.0	**0.19**	0.19	*0.20*	**0.12**	0.12	*n.d.*	0.59
2005	61.8	**0.22**	0.19	*0.24*	**0.13**	0.11	*0.14*	0.71

^a^Mean is calculated in positive samples, excluding values < LOD

^b^1 extreme value of approx. 700 ng/mL was excluded as outlier

^c^data from Zimmerli and Dick (1995)

n.d. not determined (2′R-OTA occurrence in males 50.0%)

Generally, higher levels were determined in male compared to female study participants. This was especially observed for the mean values at all measuring points (Table 2) and all means in Ticino (Table 4), while for the median values, the difference was less pronounced, and for the 2005 results no difference in the median between the sexes was observed (Table 2). A z-test of the 2019 phase I and 2005 results including the 2005 TI + samples confirmed that the values for males were significantly higher compared to females (p < 0.05).

Overall, the OTA serum levels in Switzerland were quite stable over the years, but higher levels were observed in Ticino compared to other Swiss regions and generally also for male compared to female donors. No plausible explanation can be given for these observations. During the recent decades, the OTA serum levels did not decrease significantly for any region or sex, which may be due to the fact that the OTA intake mainly results from the sum of small OTA amounts in staple foods consumed in important quantities.

#### Comparison with other studies

An important number of studies on OTA concentration in human serum or plasma has been published worldwide in the last years. Many of them revealed divergent results as described in recent reviews by Arce-López et al. (2020), Escrivá et al. (2017) and Soto et al. (2016).

However, in almost all studies there is a high percentage of blood samples being OTA positive, proving high human exposure to this mycotoxin.

In comparison with recent studies from other European countries published on healthy adults, the serum OTA levels in the present work were similar to the results of 50 blood samples in Germany reported by Osteresch et al. (2017) and Cramer et al. (2015) with a mean concentration of 0.207 ng/mL (100% > LOD, Osteresch et al.) and 0.21 ng/mL (100% > LOD, median 0.21 ng/mL, Cramer et al.). For comparison, it is important to keep in mind that the concentration ratio of OTA in serum to whole blood is around 2:1 (Korn et al. 2011; Zimmerli and Dick 1995). Also, the OTA results published by Märtlbauer et al. (2009) for 102 serum samples with a mean of 0.37 ng/mL (Germany, 98% > LOD, median 0.33 ng/mL) revealed results close to those in our study in terms of occurrence as well as concentration levels.

In contrast, the results found in our study were higher than those published for the Czech Republic by Malir et al. (2006) with an OTA mean value of 0.28 ng/mL (n = 2206, serum, 94% > LOD, median 0.2 ng/mL). Also, for two studies in Germany lower concentration levels were observed: Rosner et al. (2000) reported a mean of 0.27 ng/mL in serum (n = 927, 98% > LOD, median 0.23 ng/mL) and Muñoz et al. (2010) published similar results with a mean of 0.25 ng/mL in a small study (n = 13, plasma, 100% > LOD, median 0.24 ng/mL).

On the other hand, there were some European studies reporting higher OTA levels than our results. For instance, a comparatively high mean OTA value of 1.09 ng/mL (n = 168, plasma, 100% > LOD) was reported for blood donors in Valencia region (Spain) by Medina et al. (2010). Palli et al. (1999) published data on serum of 137 healthy adults in Italy with a mean OTA serum level of 0.56 ng/mL (97% > LOD, median 0.48 ng/mL). The mean serum result of 1.007 ng/mL in Portugal published by Viegas et al. (2018; n = 42 male participants, 100% > LOD, median 0.756 ng/mL), finally, may also be elevated due to occupational exposure from work in the waste management. Divergent results were also reported outside Europe: As examples, higher values than observed in our study were detected in plasma from Bangladesh (Ali et al. 2018) and lower plasma concentrations in a study in China (Fan et al. 2020).

Varying OTA blood levels in different regions within the same country as in our study were also reported in the review by Soto et al. (2016) who assumed that several factors such as dietary habits and climatic conditions may have an influence.

Moreover, generally higher OTA concentrations for male compared to female donors as in our study are confirmed by several authors (Soto et al. 2016). Among others, Märtlbauer et al. (2009), Medina et al. (2010), Cramer et al. (2015) and Ali et al. (2018) reported higher values for male than for female participants, but the difference was not significant. Palli et al. (1999) observed — as in our study — significantly higher OTA values for males compared to females.

Several studies have been performed in order to elucidate correlations between further parameters and OTA levels in blood. Investigated factors were, for example, age (Fan et al. 2020; Soto et al. 2016), season (Palli et al. 1999), height (Palli et al. 1999) or body mass index (Fan et al. 2020). However, no clear correlation was found (Soto et al. 2016).

Especially the results observed for the correlation between dietary OTA intake and blood levels are contradictory: Gilbert et al. (2001) found no significant correlation between OTA levels in plasma and OTA dietary intake. They measured dietary OTA intake by analysing composite duplicate diet samples and the corresponding plasma samples. In contrast, Warensjö Lemming et al. (2020) observed associations between fibre and oat intake and blood levels, and finally, Medina et al. (2010) described a weak correlation between OTA plasma levels and beef, turkey, cold meat and bread consumption. For lack of a better option, we calculated the intake of OTA from the serum values using the Klaassen equation for the risk assessment (see below).

### 2′R-OTA-Results

#### Results and trends in Switzerland over the last 15 years

In the 2019 phase I samples, 2′R-OTA was detected in 48.1% of the serum samples with a mean concentration in positive samples of 0.23 ng/mL. This is very similar to the results of the 2005 samples with a detection rate of 50.2% and a mean of 0.20 ng/mL (Table 2). Comparable results were observed for males and females (Table 2), and in samples from Ticino the 2′R-OTA serum concentration was also close to the results for whole Switzerland (Table 4).

According to a recent food study in Switzerland (menuCH 2014–2015), coffee is consumed by 78% of the Swiss adult population at an average amount of 326 g liquid/day. Assuming that blood donors do not differ considerably in terms of coffee consumption from the average Swiss population, there is a certain percentage of coffee drinkers who have very low levels of 2′R-OTA in their blood.

In our present study, the concentration ratio OTA to 2′R-OTA ranged from values over 10 down to 0.24. In altogether 61 samples (out of 337 samples with quantifiable 2′R-OTA concentrations), the 2′R-OTA level was higher than the OTA level.

#### Comparison with other studies

The results of our study are in good correlation with those published by Cramer et al. (2015) on German adults, who reported a higher occurrence of positive blood samples (68%), which may be due to lower LOD and LOQ in their quasi-single analyte method. Their mean of 0.11 ng/mL in blood (calculated for serum 0.22 ng/mL) fits perfectly to our data. However, the data published by Sueck et al. (2019b) for 16 blood samples from coffee drinkers in Germany with mean values of 0.058–0.072 ng/mL (calculated for serum 0.116–0.144 ng/mL) during coffee consumption were lower than our results. In contrast, in the study published by Viegas et al. (2018) 2′R-OTA was found in 81% of the blood serum samples from waste management workers in Portugal with a mean of 0.334 ng/mL (n = 42).

Cramer et al. (2015) observed slightly higher results in blood for male coffee drinkers (mean 0.13 ng/mL, calculated for serum 0.26 ng/mL, n = 19) than for female (mean 0.09 ng/mL, calculated for serum 0.18 ng/mL, n = 15). This is not confirmed here, and the sex differences observed in our study were small (mean in positive samples for females: 0.24 ng/mL, for males: 0.22 ng/mL). Cramer et al. (2015) observed also a great variability for the OTA-to-2′R-OTA concentration ratio in human blood and reported several samples where the 2′R-OTA content exceeded the OTA level, which is in accordance with our observations.

### Results for CIT, DH-CIT and OTα

#### CIT-Results and comparison with other studies

The presence of CIT in biological fluids has been documented in some studies, although biomonitoring data in human blood, plasma or serum are still scarce (Ali and Degen 2019; Silva et al. 2020). With the analytical method applied in the current study, lower LOD and LOQ for CIT were achieved compared to most other studies (Ali et al. 2018; Blaszkewicz et al. 2013; Malir et al. 2019; Osteresch et al. 2017; Ouhibi et al. 2020).

Nevertheless, CIT was detected in only 2.1% of the 2019 phase I Swiss serum samples in quantifiable amounts. The mean of the positive samples was 0.09 ng/mL and thus comparatively low. A low CIT occurrence was also reported by Viegas et al. (2018) who did not find CIT in any of the 42 serum samples from waste management workers in Portugal (no LOQ or LOD given). This is in line with Osteresch et al. (2017) with no positive CIT results in 50 blood samples in Germany; however, a high LOD (0.066 ng/mL) and LOQ (0.25 ng/mL) were reported in their study; consequently, several samples with low CIT level might not have been detected. With the same multi-mycotoxin method, no CIT was found in samples of 1096 blood serum samples of Swedish adolescents (Warensjö Lemming et al. 2020).

In contrast, in the first study for healthy adults performed 2013 in Germany, CIT was detected in all 8 analysed plasma samples at a level of 0.10 to 0.25 ng/mL (Blaszkewicz et al. 2013). In a further study from the same group a similar work-up but without enzymatic cleavage revealed again important amounts of CIT (n = 104, 90% > LOD, mean 0.345 ng/mL, median 0.22 ng/mL) in the plasma of young healthy adults in Bangladesh (Ali et al. 2018). CIT was also detected by Ali and Degen (2019) in plasma samples of two volunteers in Germany on their normal diet during repeated blood sampling (mean 0.27 and 0.47 ng/mL, respectively). In sum, there are widely divergent studies ranging from 100 to 0% positive samples. Although human studies with a high ratio of positive CIT samples worked with plasma, the detection of CIT in pig serum even before exposition may indicate the suitability also of serum as matrix to monitor CIT (Tkaczyk et al. 2021).

#### DH-CIT-Results and comparison with other studies

In 2019 phase I samples, DH-CIT, a metabolite of CIT, was detected in trace amounts in 4.4% of the serum samples, but only in 1 sample in a quantifiable amount (0.49 ng/mL). It should be noted that the only sample with a DH-CIT level above the LOQ is also the sample with the highest CIT level (5.70 ng/mL), while in all other quantifiable CIT samples not even traces of DH-CIT were observed.

There are only few studies reporting positive findings for DH-CIT: Ali et al. (2018) detected DH-CIT in 85% of 104 plasma samples from young Bangladesh adults (mean 0.38 ng/mL, median 0.31 mL/mL). The same group also found positive DH-CIT results in plasma for an Asian person during a 7-week study (mean 0.28 ng/mL) and a German during a 7-day study (mean 0.96 ng/mL), both with normal diet (Ali and Degen 2019).

In contrast, DH-CIT was not measurable in plasma samples from a citrinin kinetic study by the same group (applying a very similar sample preparation procedure as in Ali and Degen (2019)) in spite of a very low LOD (0.02 ng/mL) and LOQ (0.06 ng/mL) while in the same samples CIT was detected in important concentrations after oral ingestion (Degen et al. 2018). In several further studies, DH-CIT was not detected in serum samples (Viegas et al. 2018; Warensjö Lemming et al. 2020), plasma (Malir et al. 2019) or blood samples (Osteresch et al. 2017). This may partly be due to high LODs (0.27 ng/mL in Osteresch et al. (2017); 0.2 ng/mL in Malir et al. (2019)), but also in plasma or serum of pigs exposed to CIT only trace levels of DH-CIT were detected (Meerpoel et al. 2020; Tkaczyk et al. 2021).

#### OTα-Results, comparison with other studies and modified work-up with enzymatic cleavage

The metabolite of OTA, OTα, was detected in one single sample out of 700 2019 phase I samples and only in trace amounts.

This is in line with several studies, where OTα was not found in serum samples (Viegas et al. 2018; Warensjö Lemming et al. 2020) or blood (Osteresch et al. 2017). All these methods use dried sample spots and the sample preparation procedure reported by Osteresch et al. (2017) with low LOD of 0.014 ng/mL for serum and blood. Fan et al. (2019) used a different work-up with acid acetonitrile precipitation for plasma samples, but did not observe any findings above the LOQ of 0.05 ng/mL either.

Positive OTα samples were reported by others (Ali et al. 2014, 2018; Muñoz et al. 2010), for plasma samples applying a liquid-liquid extraction after enzymatic treatment with β-glucuronidase/arylsulfatase: In 2018, they observed 98% of positive samples (n = 104) with a mean of 0.38 ng/mL and a median of 0.33 ng/mL, and in 2014 95% of the 64 samples were positive with a mean of 0.28 ng/mL and a median of 0.25 ng/mL. Muñoz et al. (2010) published the first study on the presence of OTα in human plasma and detected OTα in all 13 samples in Germany with a mean of 0.95 ng/mL and a median of 1.14 ng/mL. They observed far lower levels when analysing samples without preceding enzymatic treatment (mean 0.09 ng/mL and median 0.08 ng/mL).

Based on this study, nine serum samples with elevated OTA level (0.66–1.60 ng/mL) were selected and submitted to the procedure for enzymatic cleavage described by Muñoz et al. (2010) before sample preparation. Despite the enzymatic treatment applied, no OTα content > 0.1 ng/mL (LOD) was detected in any of these samples. This is in clear contrast to Muñoz et al. (2010) who observed an important increase in their plasma samples after enzymatic cleavage.

### Comparison of 2019 phase I and phase II results

Out of the 700 blood donors from 2019 phase I, 240 volunteers could be sampled again between 2 and 9 months after the first donation (2019 phase II samples, Table 1).

#### OTA 2019 phase I versus phase II

In both samplings, around 99% of the samples revealed a quantifiable amount of OTA with comparable mean values and medians (Table 2). With the Wilcoxon signed rank-sum test, no significant difference between the phases was observed for the paired samples (p = 0.65); thus, a normal fluctuation can be assumed. OTA levels of ≥ 1.0 ng/mL at both samplings were only observed for 6 of the 240 samples. In none of the samples, the OTA level decreased between the 2 samplings to an extent, which would not be compatible with the half-life of OTA of around 35 days determined in plasma (Studer-Rohr et al. 2000).

**Fig. 2:**
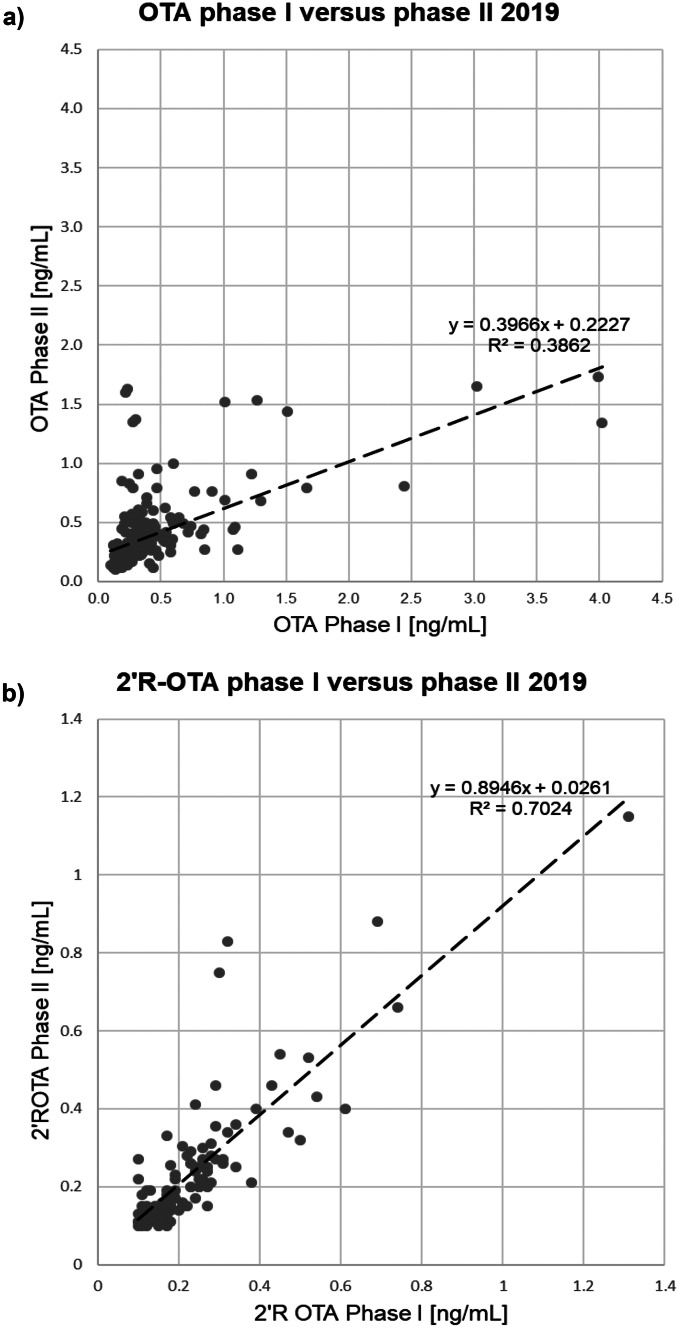
Correlation between serum levels of blood donors in Switzerland 2019 for phase I and phase II (2–9 months apart), **a**) OTA (236 subjects ≥ LOQ at both sampling points), **b**) 2′R-OTA (108 subjects ≥ LOQ at both sampling points)

The correlation between 2019 phase I and II OTA results of the individual samples is shown in Fig. 2a. Although any correlation should be evaluated with care due to the widely differing sampling intervals, the absence of a stringent association is obvious and the corresponding samples generally do not have a constant level. This is despite the fact that individual differences in metabolism are eliminated with repeated sampling of the individual. Consequently, higher or lower OTA values measured in a single serum sample do not necessarily reflect the long-term OTA level of a person.

Palli et al. (1999) re-sampled 68 individuals after one year and observed practically no correlation in the analysed samples. Only two subjects (2.9%) had OTA levels of > 1 ng/ml on both occasions. Märtlbauer et al. (2009) investigated samples from 9 individuals at 6 times at irregular measuring intervals during a period of altogether 6.5 years. They observed that although the individual values varied considerably, the mean und median contamination of all positive samples was quite constant throughout the years, which is in agreement with our data.

#### 2′R-OTA 2019 phase I vs phase II

In both samplings, around 50% of the samples revealed a quantifiable amount of 2′R-OTA with comparable mean values of positive samples (Table 2). For 4 of the 240 samples, 2′R-OTA levels of ≥ 0.5 ng/mL at both samplings were observed. Sueck et al. (2019b) found strong indications for an unexpectedly long biological half-life of 2′R-OTA of at least seven months. In only 3 of our samples, the 2′R-OTA level decreased to such an extent that the results would not be compatible with this assumed long half-life of 2′R-OTA in blood.

Figure 2b shows the correlation between the 2′R-OTA levels at the two samplings (2–9 months difference in time). The graph reveals obvious differences between the OTA (Fig. 2a) and the 2′R-OTA correlation: The correlation for 2′R-OTa is higher and the slope of the regression line is far closer to 1, which may indicate levels that are more constant. This could be due to a longer biological half-life and/or more constant intake, for example, with the daily coffee consumption.

For CIT, DH-CIT and OTα, the number of positive 2019 phase II samples was too low to compare any results.

### Risk assessment of the OTA-results

As mentioned above, OTA was recently re-evaluated by EFSA (EFSA 2020). For the characterisation of non-neoplastic effects, a benchmark dose lower confidence limit for an extra risk of 10% (BMDL_10_) value of 4.73 µg/kg bw per day was calculated from the kidney lesions observed in pigs (Krogh et al. 1974). Using a MOE of 200 (default factor 100, factor 2 for extrapolation from subchronic to chronic exposure), this corresponds to a target value of 23.7 ng/kg bw per day. To characterise neoplastic effects, a BMDL_10_ value of 14.5 µg/kg bw per day was determined from the kidney tumours observed in rats (NTP 1989). Applying a MOE for genotoxic-carcinogenic substances of 10′000, this corresponds to a target value of 1.45 ng/kg bw per day. In interpreting the MOE for neoplastic risks, EFSA considered that the MOE of 10′000 for substances that are directly genotoxic and carcinogenic may not be appropriate in this case, as the evidence for a direct interaction of OTA with DNA is inconclusive (EFSA 2020). However, in the absence of clarified mechanisms of action for the genotoxicity and carcinogenicity of OTA, EFSA concluded that a default MOE of 10′000 should be applied to the BMDL_10_ value of 14.5 µg/kg bw per day for neoplastic effects (kidney tumours) in the rat.

The daily dietary intake of OTA can be calculated from the measured serum level by using the equation of Klaassen (1986): *k*_*0*_ = *Cl*_*p*_* x C*_*p*_*/A,* where k_0_ is the daily intake (ng/kg bw per day), Cl_p_ the plasma filtration rate (mL/kg bw per day), C_p_ the serum concentration (ng/mL) and A the gastrointestinal absorption (see also Coronel et al. (2010)).

Three different values are commonly applied for Cl_p_. The first value for Cl_p_ is 0.033 mL/min, which corresponds to 0.67 mL/kg bw per day. It is based on the renal filtration rate of inulin (140 mL/kg bw per hour) and the free OTA fraction in plasma (0.02%), which allowed the calculation of the renal filtration for OTA in humans (Hagelberg et al. 1989). The second value for Cl_p_ is 0.048 mL/min that corresponds to 0.99 mL/kg bw per day. It originates from a human study with one volunteer who ingested tritium-labelled OTA (Studer-Rohr 1995). The third value for Cl_p_ is 0.11 mL/min corresponding to 2.26 mL/kg bw per day. It is based on the same data by Studer-Rohr (1995) but was derived by linear regression in a later evaluation (Studer-Rohr et al. 2000).

A 50% gastrointestinal absorption was used in this assessment based on studies by Galtier et al. (1979, 1981) and Hagelberg et al. (1989) on different species. The following models were used: A) k_0_ = 0.67 × C_p_/0.5 = 1.34 × C_p_ (Hagelberg et al. 1989), B1) k_0_ = 0.99 × C_p_/0.5 = 1.97 × C_p_ (Studer-Rohr 1995) and B2) k_0_ = 2.26 × C_p_/0.5 = 4.52 × C_p_ (Studer-Rohr et al. 2000). All models are often applied, e.g., model A and B1 by Coronel et al. (2010) and the Commission for Human Biomonitoring of the German Environment Agency (2014) and model B2 by Penczynski et al. (2022). Models B1 and B2 were used by Warensjö Lemming et al. (2020). In the present evaluation, all three models are employed.

Using the target value for non-neoplastic effects of 23.7 ng/kg bw per day, the following target values for human serum were calculated: 17.69 ng/mL (model A), 12.03 ng/mL (model B1) and 5.24 ng/mL (model B2). Using the target value for neoplastic effects of 1.45 ng/kg bw per day and applying equivalent calculations resulted in the following target values: 1.08 ng/mL (model A), 0.74 ng/mL (model B1) and 0.32 ng/mL (model B2).

In this evaluation, only the results of the samples from 2019 phase I were taken into account. In the first case, all values (n = 700) were considered; in the second case only the values from Ticino (n = 100).

The target value for non-neoplastic effects was exceeded in models A and B1 by only two samples and in model B2 by three samples. All three samples came from Ticino.

The target value for neoplastic values was met by 96.1% (model A), 92.7% (model B1) or 62.0% (model B2) of all serum values. If only the serum values from Ticino were taken into account, 88.0% (model A), 82.0% (model B1) or 45.0% (model B2) of the samples were below the target value.

## Final remarks

In summary, OTA was found to be the prevalent mycotoxin in human serum samples in Switzerland followed by 2′R-OTA, while CIT, DH-CIT and OTα were of minor occurrence. Overall, the OTA serum levels in Switzerland were quite stable over the years but higher levels were observed in Ticino compared to other Swiss regions and generally also for male compared to female donors.

No significant decrease was observed for any region or sex during the recent decades, which may be due to the fact that the OTA intake mainly results from the sum of small OTA amounts in staple foods consumed in important quantities.

Relying on serum levels as a marker for OTA intake, very few individuals are at risk for non-neoplastic effects. For neoplastic effects, the risk strongly depends on the model used. About 5% of the Swiss population might be at a certain risk as the MOE is less than 10′000 in model A and B1 and 38% in model B2. For the Ticino region, however, this applies to about 10 to 20% of the population with models A and B1 and 55% in model B2. Nevertheless, applying a MOE of 10′000 is likely to be a very conservative approach for OTA, especially since EFSA (2020) concluded that the risk may be overestimated due to high uncertainty.

Serum OTA levels provide a valuable exposure estimation of population groups; nevertheless, it is still uncertain to which extent the metabolism of the individual influences the correlation between OTA intake and observed serum levels. To get further insight, a study in which analysis of the total OTA intake from diet combined with the corresponding serum levels would be very elucidating.

2′R-OTA serum levels were stable over the years, and unlike for OTA no sex- or region-specific differences were observed. It is interesting to note the discrepancy between the percentage of people consuming coffee (78%) and the ratio of 2′R-OTA occurrence in serum (approximately 50%), although the reasons for this observation are unknown.

The establishment of a certified control serum containing at least OTA, 2′R-OTA and CIT in known amounts is very desirable to verify method performance and minimise the bias of analytical results. Since OTA is a nearly ubiquitous contaminant in human serum, in the meantime interested laboratories could use readily available serum samples certified for other purposes also for OTA and share the results.

Finally, for an in-depth international comparison of the serum concentrations, a study with samples from several countries that are analysed in the same laboratory with one method would be very useful.

## Supplementary information

Below is the link to the electronic supplementary material.

Supplementary file1 (DOCX 88 KB)
