# Supplementary material for: Biomonitoring of ochratoxin A, 2′R-ochratoxin A and citrinin in human blood serum from Switzerland
Source: Mycotoxin Res. 2022 Apr 20;38(2):147–61. doi: 10.1007/s12550-022-00456-0 (PMC9038883; doi:10.1007/s12550-022-00456-0)
Supplement: Supplementary file 1 — Supplementary file1 (DOCX 88 KB) [file 12550_2022_456_MOESM1_ESM.docx]

**Electronic Supplementary Material for:**

**Biomonitoring of ochratoxin A, 2′R-ochratoxin A and citrinin in human blood serum from Switzerland**

Alexandra Jaus*, Peter Rhyn, Max Haldimann, Beat Brüschweiler, Céline Fragnière Rime, Judith Jenny-Burri, Otmar Zoller

Risk Assessment Division, Federal Food Safety and Veterinary Office (FSVO), Schwarzenburgstrasse 165, Bern 3003, Switzerland

*Corresponding author: alexandra.jaus@blv.admin.ch


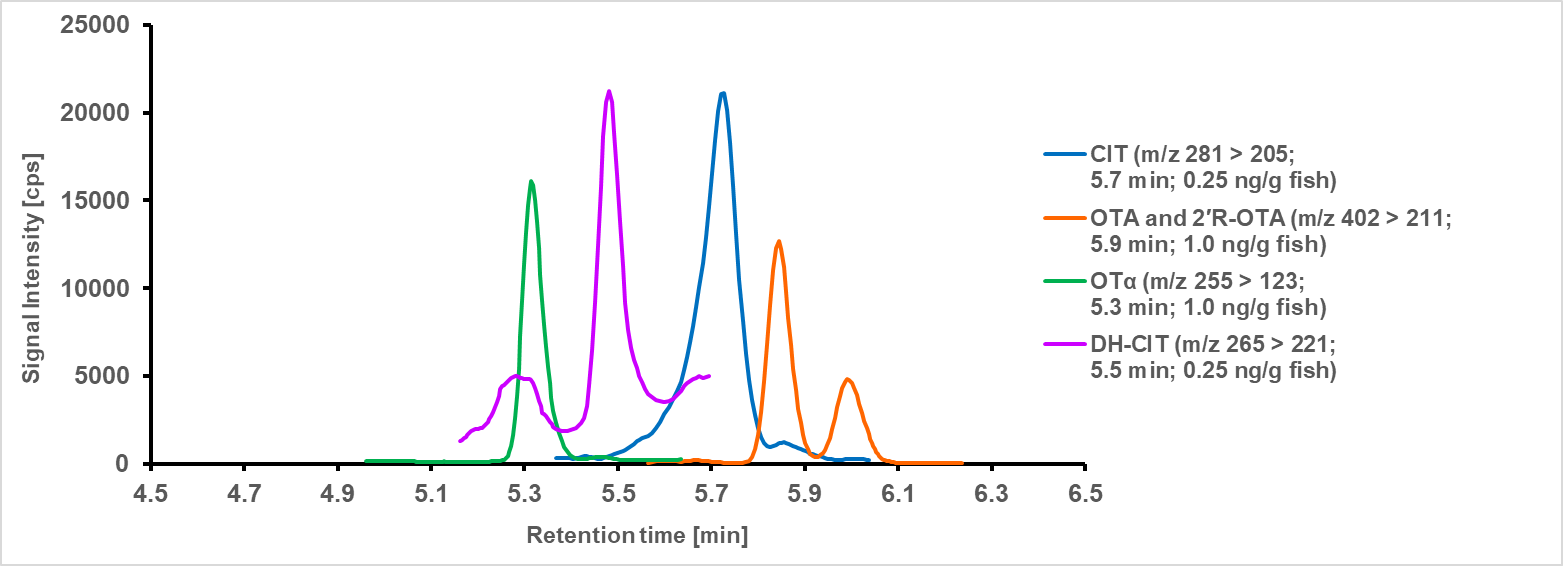


Figure S1: HPLC-MS/MS chromatogram of a serum sample spiked with OTα, DH-CIT, CIT, OTA and 2′R-OTA.

Table S1: Detailed sMRM parameters for all analytes, quantifier in bold

| Analyte | Q1 mass [m/z] | Q3 mass [m/z] | Declustering Potential [V] | Collision Energy [eV] | Expected Retention Time^a^ |
| --- | --- | --- | --- | --- | --- |
| OTA/ 2′R-OTA | 402 [M-H]^-^ | **211**  167 | -50 | -35  -40 | 5.9^b^ |
| CIT | 281 [M+MeOH-H]^-^ | **205**  249 | -90 | -31  -19 | 5.7 |
| OTα | 255 [M-H]^-^ | **123**  167 | -65 | -42  -35 | 5.3 |
| DH-CIT | 265 [M-H]^-^ | **221**  177 | -80 | -28  -35 | 5.5 |

^a^ Expected retention time used for programming the sMRM

^b^ OTA elutes just before 2′R-OTA but both substances have the same fragmentation pattern

Table S2: Validation results for all analytes

| Analyte | LOQ [ng/mL] | LOD [ng/mL] | Recovery [%]  (conc. ng/mL), n=5 | Inter-day precision  [%] n=10 | Injection repeatability high [%] n=5 | Regression coefficient (r^2^) |
| --- | --- | --- | --- | --- | --- | --- |
| OTA | 0.1 | 0.1^a^ | low 75 ± 8.2 (0.15)  high 108 ± 4.2 (0.5) | low 10.4  high 18.6 | 3.5 | 0.9933 |
| 2′R-OTA | 0.1 | 0.1^a^ | low 89 ± 14.1 (0.15)  high 106 ± 12.0 (0.5) | low 12.4  high 18.7 | 5.9 | 0.9940 |
| CIT | 0.05 | 0.02^b^ | low 95 ± 2.2 (0.1)  high 104 ± 2.7 (0.25) | low 3.6  high 5.3 | 2.8 | 0.9964 |
| OTα | 0.25 | 0.1 | low 107 ± 4.6 (0.25)  high 115 ± 6.6 (0.5) | low 10.0  high 24.0 | 15.5 | 0.9921 |
| DH-CIT | 0.25 | 0.1 | low 92 ± 7.4 (0.25)  high 89 ± 15.7 (0.5) | low 10.9  high 13.2 | 8.7 | 0.9953 |

^a^ S/N ratio of the next lower standard did not exceed S/N ratio >3 any more

^b^ calculated from the lowest standard 0.05 ng/mL

Sensitivity: for LOQ (LOD) the lowest calibration point with S/N ratio >10 (>3) is given.

Regression coefficients: for a typical calibration with 7 points in the range from 0.0 to 2.0 ng/mL.

The recovery results, injection repeatability and inter-day precision are reported with their RSD for the quantifier transition and are given in %.
